# Sequence-specific detection and analysis of nucleic acids via hybridization on magnetic beads and MALDI-TOF MS readout

**DOI:** 10.1007/s00216-025-06193-4

**Published:** 2025-11-13

**Authors:** Susanne Dietrich, Jessica Beyerl, Susanna Oswald, Anna-Cathrine Neumann-Cip, Andreas Wieser, Christoph Haisch

**Affiliations:** 1 TUM School of Natural Sciences, Technical University of Munich, Munich, Germany; 2 Fraunhofer Institute for Translational Medicine and Pharmacology ITMP, Immunology, Infection and Pandemic Research, Munich, Germany; 3 Institute of Infectious Diseases and Tropical Medicine, LMU University Hospital, LMU Munich, Munich, Germany; 4 German Center for Infection Research (DZIF), Partner Site Munich, Munich, Germany

**Keywords:** MALDI-TOF MS, Oligonucleotides, Hybridization, Mass spectrometry, Nuclease, Digestion

## Abstract

In molecular diagnostics, the sequence-specific extraction and detection of nucleic acids remain a significant challenge. Here, we present a straightforward method that enables both the sequence-specific enrichment and subsequent analysis of nucleic acids using MALDI-TOF MS as a readout. Functionality and performance were tested successfully by using magnetic beads coated with DNA, which is complementary to the target nucleic acid, as a substrate. For sequence-specific extraction, hybridization of the target nucleic acid was performed, and subsequently, the single-stranded overhang was digested. The analysis was performed by *matrix-assisted laser desorption/ionization time-of-flight mass spectrometry* (MALDI-TOF MS) without further elution. The specificity was determined by hybridization of DNA oligonucleotides, which differ in up to four nucleobases in the middle of the strand. Two mutations led to a significant signal reduction. Mutated, but hybridizing strands can be distinguished from an entirely complementary strand by their mass. The specificity is higher for mutations located in the middle of the target strand. By employing single-strand-specific nucleases, we successfully digested a single-stranded overhang, resulting in the most intense signal corresponding to the blunt end. Knowing the mass of the immobilized oligonucleotide, the mass of the searched complementary mass can be calculated. Therefore, even with more than one digestion product, the searched oligonucleotide can be determined by its mass. The integration of hybridization selectivity, minimal sample preparation, short measurement time, and mass accuracy results in a robust and precise method for detecting specific sequences.

**Graphical abstract:**

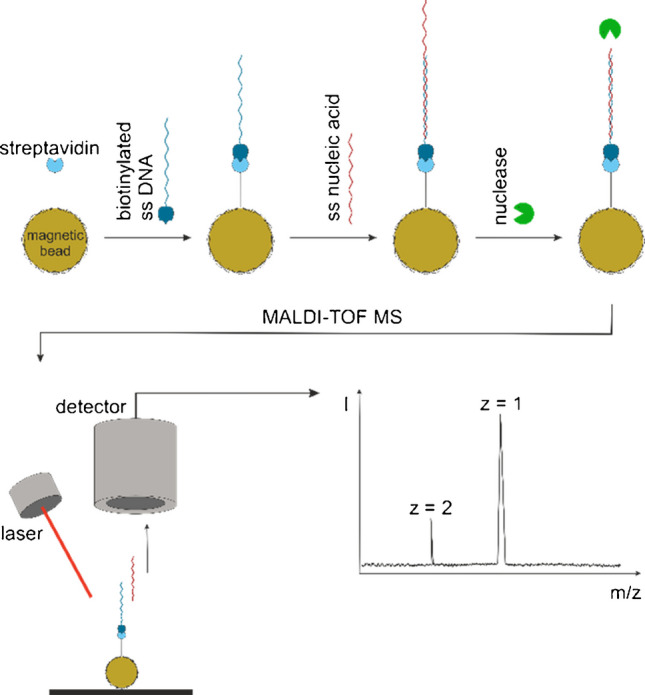

**Supplementary Information:**

The online version contains supplementary material available at 10.1007/s00216-025-06193-4.

## Introduction

Nucleic acid detection and analysis are fundamental to numerous fields, including molecular biology, diagnostics, forensic science, and biomedical research [1–3]. The specificity and sensitivity of detection methods are paramount, particularly for pathogen identification, genetic analysis, and biomarker discovery [4–6]. Current detection techniques, such as polymerase chain reaction (PCR) [7], loop-mediated isothermal amplification (LAMP) [8], microarrays [9], and fluorescence-based assays [10], rely heavily on hybridization techniques that utilize sequence-specific probes to identify target sequences. These methods have been pivotal in advancing our understanding of genetic material, yet they often require complex procedures and extensive sample preparation [11]. PCR-based methods, for example, are prone to contamination, require extensive sample preparation, and often exhibit temporal variability in amplification efficiency due to enzyme degradation or reaction conditions [12]. This renders these methods less appropriate for high-throughput screening and real-time monitoring of bacterial growth [13]. Hybridization-based detection strategies have been developed to improve sequence specificity, utilizing complementary oligonucleotide probes to capture target sequences selectively. Advances in hybridization techniques have led to the integration of various signal amplification techniques, such as enzyme-assisted cleavage and nucleic acid-templated reactions, improving detection limits and selectivity [12, 14]. However, these approaches often require additional labeling steps or enzymatic reactions, which can introduce complexity, variability, and potential biases. Moreover, temperature fluctuations, ionic strength, and secondary structures within the nucleic acid sequences can influence hybridization kinetics and binding efficiency, leading to signal variations [15]. Despite advancements, current nucleic acid detection methods have several limitations. Many require complex workflows involving precise thermal cycling and multiple reagents. The need for amplification steps or enzymatic reactions further increases processing time [11]. Additionally, techniques like fluorescence-based assays and CRISPR systems can be costly due to their reliance on specialized reagents. Magnetic beads are particularly suitable for extracting nucleic acid biomarkers from biological samples [3, 16, 17]. Depending on the target detection strategy, different bead functionalities can be chosen. Sequence-specific bead functionalities are required to analyze specific, short RNA sequences without the need for high concentrations for detection [18].

Recent research has explored mass spectrometry (MS) as an alternative nucleic acid detection platform, particularly *matrix-assisted laser desorption/ionization time-of-flight* (MALDI-TOF) MS. This technique enables label-free detection of nucleic acids based on mass-to-charge ratio analysis, offering high-throughput capabilities with minimal sample preparation [19]. MALDI-TOF MS has been successfully applied for genotyping and microbial identification [20]. However, it often lacks sufficient sequence specificity without additional hybridization steps [15, 19]. Temporal variations in ionization efficiency, matrix composition, and sample degradation can impact MALDI-TOF MS performance, necessitating optimized protocols for reliable nucleic acid detection [13, 21, 22].

Integrating hybridization techniques with MALDI-TOF MS advances nucleic acid detection [23]. Tang et al*.* demonstrated the use of streptavidin-coated magnetic beads with immobilized biotinylated single-stranded DNA to capture a complementary oligonucleotide, followed by MALDI-TOF MS readout [24]. By immobilizing a complementary short oligonucleotide sequence on a magnetic particle, only the target analyte hybridizes, allowing for precise sequence-specific detection [11]. MALDI-TOF MS further enhances this process by providing rapid and automated analysis of nucleic acid fragments. This approach also combines the specificity of hybridization with the high-throughput potential, the sensitivity, and the accuracy of MALDI-TOF MS, but focuses on the analysis of punctual mutations and the digestion process of single-stranded extensions.

## Experimental section

### Chemicals and reagents

The Oligonucleotide Calibration Standard was supplied from Bruker Daltonik GmbH (Bremen, Germany). Dynabeads™ M-280 Streptavidin, Phosphate-Buffered Saline (PBS, 10X, RNase-free), and S1 Nuclease (100 U µL^−1^) were purchased by Thermo Fisher Scientific (Schwerte, Germany). 3-hydroxypicolinic acid (3-HPA) (≥ 99.0%), diammonium hydrogen citrate (DAC) (≥ 99.0%), and acetonitrile (ACN) (≥ 99.9%) were from Sigma-Aldrich (Taufkirchen, Germany). The solvent ethylenediaminetetraacetic acid disodium salt dihydrate (EDTA) (≥ 99%) and 2-propanol (≥ 99.5%) were purchased from Carl Roth GmbH – Co. KG (Karlsruhe, Germany). Nuclease-Free Water, MgCl_2_ (25 mM), and PCR Buffer (10x) were supplied by Qiagen (Hilden, Germany). The nucleases P1 (100,000 U mL^−1^), Exonuclease T (ExoT) (5000 U mL^−1^), and Mung Bean (MB) (10,000 U mL^−1^) with MB Nuclease Reaction Buffer were supplied from New England Biolabs (Frankfurt am Main, Germany). All oligonucleotides were purchased from metabion (Planegg, Germany). All solvents and reagents were at least analytical reagent grade.

### Sample preparation

The Dynabeads were prepared by vortexing and mixing them with an equal volume of PBS (25 µL). The beads were separated with a magnet, and buffer was exchanged (MagSep/BufEx) thrice with 50 µL PBS. The particles were resuspended in 50 µL PBS. Biotinylated DNA (1 µL) was diluted in 49 µL H_2_O and added to the bead solution. After mixing for 10 min, MagSep/BufEx was performed thrice with 100 µL PBS. The particles were resuspended in a solution containing 37 µL H_2_O, 5 µL PCR buffer, and 8 µL Mg^2+^ solution. Complementary DNA (cDNA, 1.5 µL) was added, mixed at the respective melting temperature of the immobilized sequence (3 min, 1000 rpm), and then cooled to 35 °C. The particles were washed by MagSep/BufEx thrice with 50 µL tempered H_2_O and resuspended in 25 µL H_2_O. If a longer cDNA was used and a digestion step was needed to remove the overhang, the particles were resuspended in Mung Bean buffer. Nuclease (1 µL) was added, and the solution was shaken for the respective time (30 °C, 1000 rpm). To stop the digestion process, 3 µL EDTA (0.5 M) was added at room temperature (RT). After 3 min, the particles were washed by MagSep/BufEx five times with 50 µL H_2_O and resuspended in 25 µL H_2_O. For the elution of the biotinylated oligonucleotide, the solution was heated to 80 °C.

Used sequences for mutation analysis:

- angel: Biotin – 5′ – TTC AGT AGT CTT TTT ACC AG – 3′ (T_m_ = 52 °C)
- 0 mutations: 3′ – AAG TCA TCA GAA AAA TGG TC – 5′ – FITC (T_m_ = 52 °C)
- 2 mutations: 3′ – AAG TCA TCA TCA AAA TGG TC – 5′ – FITC (T_m_ = 52 °C)
- 4 mutations: 3′ – AAG TCA TCT TCG AAA TGG TC – 5′ – FITC (T_m_ = 54 °C)
- 2 mutations at 3′: 3′ – GCG TCA TCA GAA AAA TGG TC – 5′ – FITC (T_m_ = 56 °C)
- 2 mutations at 5′: 3′ – AAG TCA TCA GAA AAA TGG GA – 5′ – FITC (T_m_ = 52 °C)
- 4 mutations at 3′: 3′ – GCC GCA TCA GAA AAA TGG TC – 5′ – FITC (T_m_ = 58 °C)
- 4 mutations at 5′: 3′ – AAG TCA TCA GAA AAA TTC GA – 5′ – FITC (T_m_ = 50 °C)
- 6 mutations at 3′: 3′ – GCC GAT TCA GAA AAA TGG TC – 5′ – FITC (T_m_ = 56 °C)
- 6 mutations at 5′: 3′ – AAG TCA TCA GAA AAT GTC GA – 5′ – FITC (T_m_ = 52 °C)

Used sequences for digestion analysis:

- angel: Biotin – 5′ – GTG AGG ATC GAT TCC A – 3′ (T_m_ = 48 °C)
- cDNA: 3′ – CAC TCC TAG CTA AGG TAG AAT GTC TGA CTT GG – 5′ (T_m_ = 72 °C)

### MALDI-TOF MS analysis

For MALDI-TOF MS measurements, which were performed with an Autoflex Speed mass spectrometer (Bruker Daltonik GmbH, Bremen, Germany), a polished ground steel MALDI target plate (Bruker Daltonik GmbH, Bremen, Germany) was used. Each sample was spotted directly onto the MALDI target (1 µL), dried at RT, and overlaid with 1 µL of MALDI matrix (3-HPA: saturated 3-hydroxypicolinic acid in 10 mg mL^−1^ DAC in a 1:1:1 solution of ACN:2-propanol:H_2_O), which was again allowed to dry. The MS is equipped with a 1 kHz smartbeam-II laser and controlled by the FlexControl software (version: 3.4.135.7, Bruker Daltonik GmbH, Bremen, Germany). All spectra were recorded in the negative linear mode in the mass range between 2000 Da and 20,000 Da. The parameter settings were optimized as follows: ion source 1: 19.50 kV, ion source 2: 18.35 kV, pulsed ion extraction time: 330 ns. Gain and laser power were set to the manufacturer’s recommended values for detecting oligonucleotides.

An external standard was used for instrument calibration, containing three oligonucleotides (12-, 20-, and 30-mer) with masses between 4000 Da and 10,000 Da (Oligonucleotide Calibration Standard, Bruker Daltonik GmbH, Bremen, Germany). All spectra are based on the average of 10,000 pulses accumulated from different points of the target. Data was processed using the FlexAnalysis software (version: 3.4.76, Bruker Daltonik GmbH, Bremen, Germany).

## Results and discussion

### Sequence-specific hybridization on magnetic beads

Magnetic beads coated with streptavidin were used to extract a specific nucleic acid sequence from a mixture. For this, biotinylated complementary DNA single strands, which serve as a hinge, were coupled as ligands to the surface of the magnetic particles. The biotinylated DNA sequence consists of 20 nucleotides (n_N_ = 20). Synthetic DNA could be hybridized with the modified particles in a sequence-specific manner. Elution of the DNA from the particle surface was not necessary for detection, as the intermolecular interactions of the DNA hybrid are disrupted during the ionization process, whereas the streptavidin–biotin bond remains intact. Detection of the biotinylated ligand DNA by MALDI-TOF MS is only possible after elution at temperatures exceeding 70 °C, at which streptavidin denatures.

**Fig. 1:**
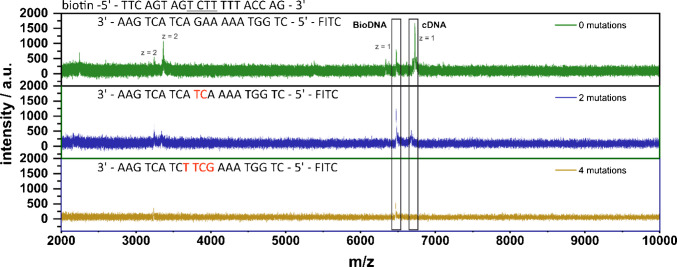
Mass spectrum of oligonucleotides after elution by heating to 80 °C. Oligonucleotides with zero, two, and four mutations (cDNA) were hybridized to immobilized oligonucleotides (BioDNA)

To investigate sequence-specific hybridization, we focused on single-point mutations, where one single nucleotide is replaced by another. Less hybridization upon a mutation signifies a more specific system. If hybridization takes place despite mutation, our MS readout still allows to differentiate the mutated strand from the entirely complementary strand by its mass. The specificity was investigated by introducing DNA fragments of the same length, which differ in up to four nucleotides in the middle of their sequence, for hybridization. Hybridization occurred at 52 °C, the specific hybridization temperature of the immobilized sequence. To evaluate reproducibility, the experiments were repeated five times. The DNA fragments were tested separately before assessing the hybridization specificity in the mixture of all sequences. Figure 1 shows the spectra with zero (top), two (middle), and four (bottom) mutations in the sequence. In all spectra, a signal of the biotinylated strand at 6480 Da is detectable after elution, showing that the immobilization worked and therefore a strand for hybridization was available. The oligonucleotide with four mutations could not be detected in the eluate by MS (Fig. 1, bottom), meaning hybridization to the ligand DNA was not possible. The signal of the oligonucleotide with two mutations (Fig. 1, middle) retained only (22 ± 10) % of the intensity of the signal from the oligonucleotide without mutations (Fig. 1, top). From the mixture of DNA fragments with various degrees of mutations, only the sequence complementary to the ligand DNA was isolated and detected by MALDI-TOF MS, indicating high sequence specificity, especially among competing DNA fragments. As expected, lower temperatures (T < 40 °C) led to non-specific accumulation of the slightly mutated DNA fragments and thus lower specificity. Higher temperatures (T > 70 °C) resulted in lower signals in the mass spectrum due to denaturation of the streptavidin and, thus, elution of the ligand DNA. Further investigations showed that also a sequence with three mutations (3′ – AAGTCATCACTCGAAATGGTC – FITC – 5′) does not hybridize with the ligand DNA. To further confirm successful hybridization, the oligonucleotides were additionally labeled with a fluorescent dye (Fluorescein-5,6-isothiocyanate, FITC) and analyzed by fluorescence microscopy. The corresponding images are shown in the supporting information S1.

In addition to mutations in the middle part of the sequence, mutations at the strand ends, both proximal to the particle surface and distal from it, were investigated. Oligonucleotides with two, four, and six mutations were tested at the 3′ and 5′ ends. Unsurprisingly, hybridization also occurs with six mutations, as the mutated sequence part is spatially distant and thus does not influence the hybridization of the complementary part. No difference was observed in the effect of mutations, regardless of whether they were proximal to the particle surface or distal from it.

While mutations in the middle of the sequence reduce hybridization for two mutations and prevent it totally for three mutations, mutations at the end of the strand, which do not influence hybridization, can only be detected by the mass difference. Mutations spread, with gaps in between, have not been tested as control; we expect a low hybridization probability.

### Enzymatic digestion of protruding nucleotide sequence residues

**Fig. 2:**
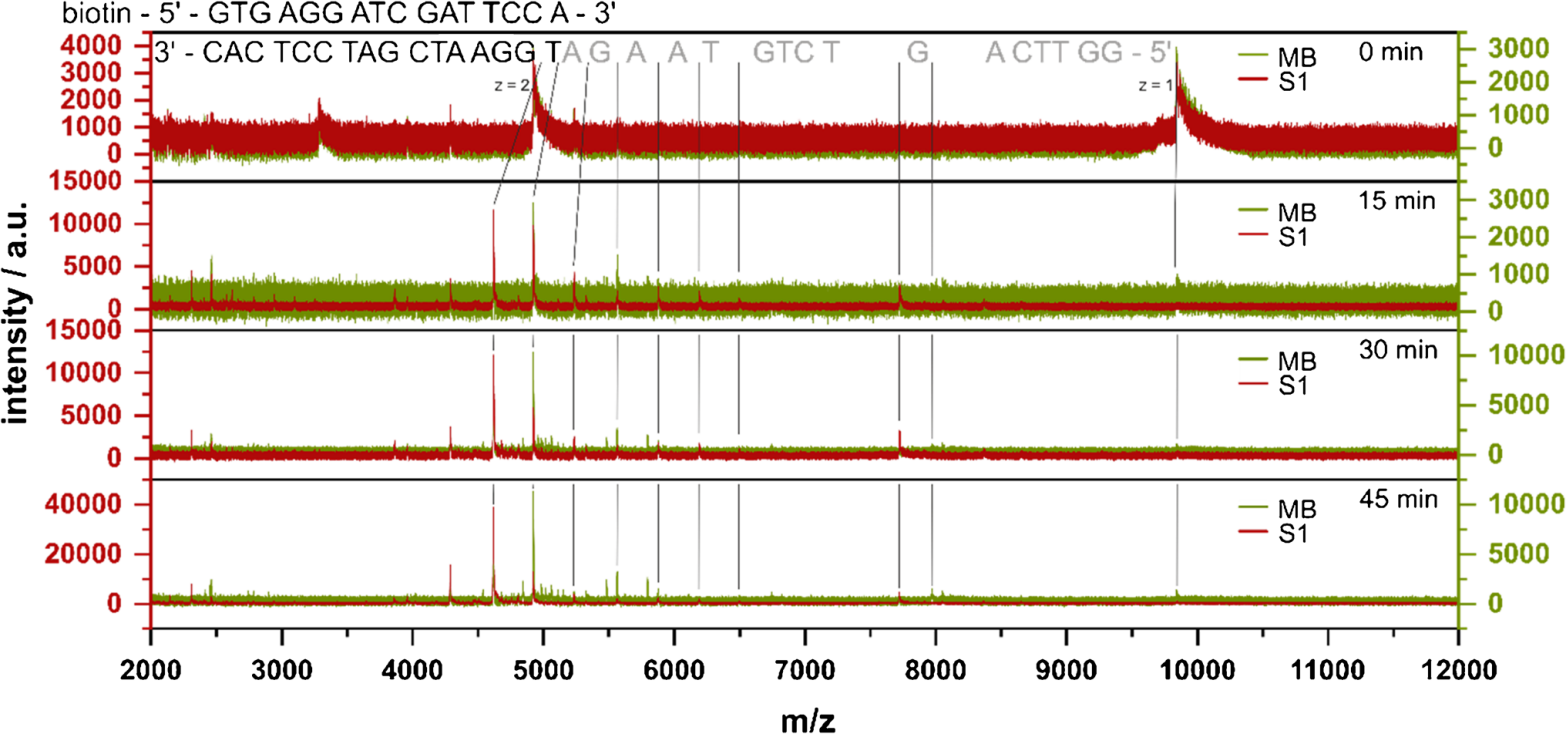
Comparison of S1 and MB digestion. The sequence of the double-stranded region is written in black, and the sequence of the single-stranded extension is in grey. Samples were taken after zero, 15, 30, and 45 min of digestion

The sequences with fragment lengths of around 20 bases represent only a minute section of bacterial nucleic acids. They represent a compromise that satisfies mass spectrometry and sequence-specific hybridization requirements, along with a manageable hybridization temperature. For the analysis of nucleic acid fragments using MALDI-TOF MS, the length must not be too long, as the signal intensity and the measurement resolution decrease with longer chain length. On the other hand, the fragments should not be too short for sequence-specific applications, as shorter lengths can compromise the specificity and stability of the binding. Another critical point is, therefore, to show that corresponding enzymes can digest protruding ends of longer RNA or DNA fragments. To ensure that only the overhang, but not the hybridized part, is digested, single-strand-specific nucleases must be used that act at both (3′- and 5′-) ends of the RNA or DNA. Biotinylated DNA (n_N_ = 16) was immobilized on magnetic particles, and complementary DNA with a longer sequence (n_N_ = 32) was hybridized. For all four tested nucleases, P1 (endonuclease), ExoT (endonuclease), S1 (exonuclease), and MB (endonuclease), successful digestion of protruding residues of the hybridized DNA on the particle surface could be detected by MALDI-TOF MS. As already shown in the literature, MB (30,000) shows the highest selectivity towards the single strand based on their ssDNase:dsDNase activity ratios and kinetic constants, followed by nuclease S1 (10,000) [25]. Nevertheless, as shown in Fig. 2, nuclease S1 has the advantage that it digests faster than MB. The spectra show the remaining sequences, which are hybridized to the immobilized strand and not the digested nucleotides, which are washed away. After 15 min digestion with S1, no more signal of the whole strand (9839 Da) is observed. In contrast, the entire strand is still detectable after 45 min digestion with MB. Generally, S1 shows almost twice as many digestion products after 15 and 30 min of digestion as MB. After 45 min, the highest signal of S1 digestion is one nucleobase in the double-strand region (3′ – CAC TCC TAG CTA AGG – 5′, 4537 Da), whereas MB shows the most intense signal between the single-strand extension (grey part) and the double-strand (black part; 3′ – CAC TCC TAG CTA AGG T – 5′, 4841 Da). Under the conditions tested, it was impossible to generate so-called *blunt ends*, where both sequences of a hybrid have the same length, ensuring that only the exact DNA fragment complementary to the ligand DNA remains.

Despite single-strand specificity, high nuclease concentrations also led to parts of the double-strand being digested, while too low concentrations resulted in varying numbers of protruding nucleotides. Since hybridization is a dynamic process and the hybrids are not covalently bound to each other, one aspect to investigate was the varying GC content. While the base pair AT builds two hydrogen bonds, GC builds three hydrogen bonds, which means that GC-rich hybrids are more stable and have higher melting temperatures. Indeed, if the double-stranded region has a higher GC content, fewer digestion products in the ds region are observed, as shown in the supporting information S2. Another aspect to investigate was whether the hybrids could open and close easily, at least partially, at an optimal digestion temperature of 30 °C. To investigate this, the digestion process was also carried out at 20 °C and 4 °C. Even at temperatures of 4 °C, where possible opening and closing are reduced, parts of the double-strand were also digested. It can be concluded that the single-strand-specific nuclease is not fully selective to single strands and partially digests the double-stranded region as well.

**Fig. 3:**
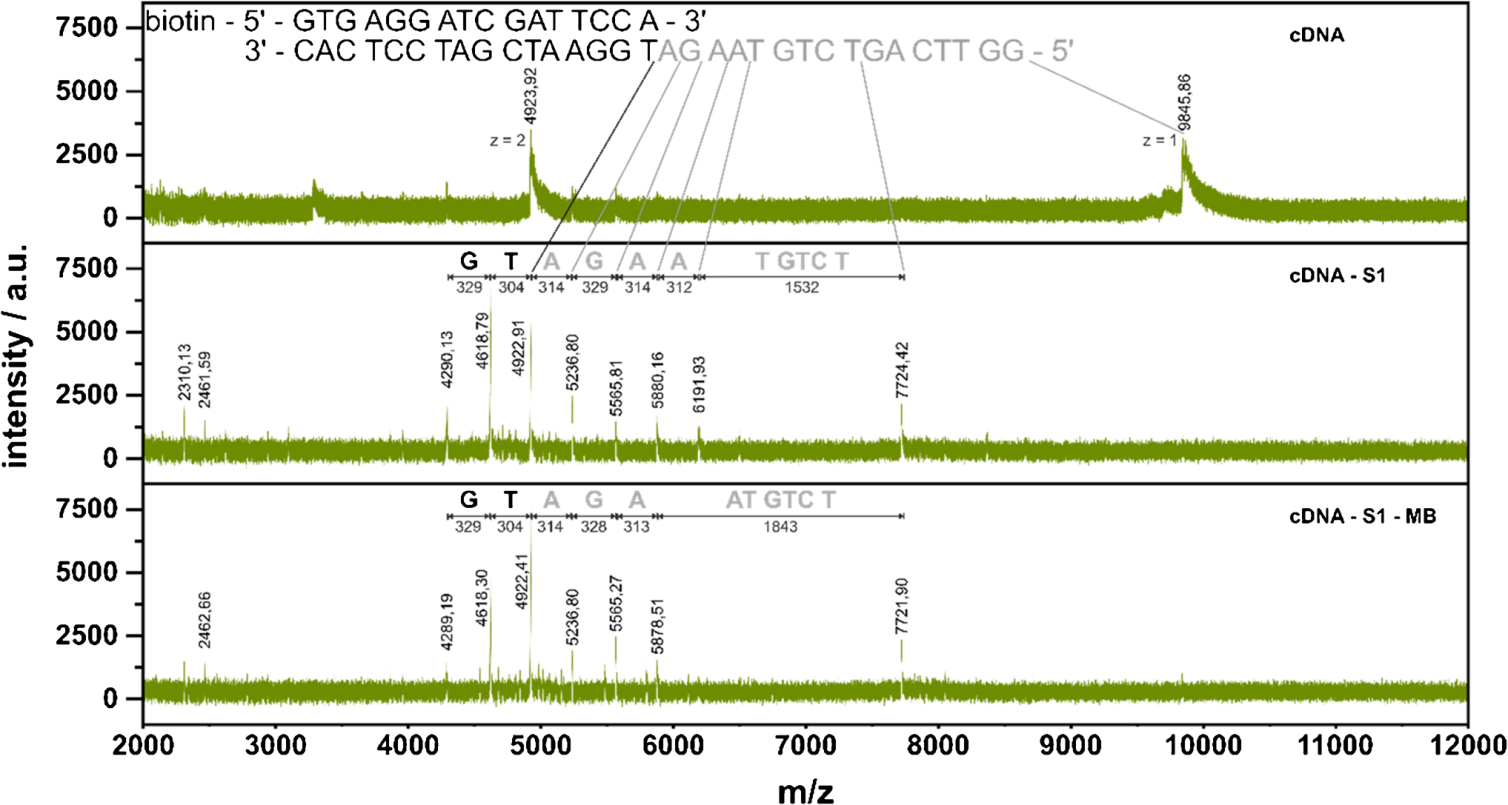
Mass spectrum of oligonucleotides before digestion (top), after the first digestion step with S1 (middle), and after the second digestion step with MB (bottom). The sequence of the double-stranded region is written in black, and the sequence of the single-stranded extension is in grey

The nucleases S1 and MB were combined to achieve both efficiency and selectivity, as shown in Fig. 3. S1 was used for the initial digestion process. After 10 min, the complementary DNA with a complete overhang (3′ – CAC TCC TAG CTA AGG TAG AAT GTC TGA CTT GG – 5′, 9839 Da) was no longer detectable. In addition to the overhang’s digestion products, two digestion products were already in the double-stranded region, including the most intense signal. The signal with the highest intensity corresponds to the sequence 3′ – CAC TCC TAG CTA AGG – 5′ (4537 Da), which is one nucleotide less than the whole double-stranded region (black part). After 10 min, S1 was exchanged with MB, and the digestion process was continued for 20 min. After this, the most intense signal corresponded to the DNA sequence in which the single-stranded overhang was completely digested (3′ – CAC TCC TAG CTA AGG T – 5′, 4841 Da).

### Realistic matrix analyses

Microparticles, as the used magnetic beads, offer a large specific surface area, which facilitates efficient binding of the immobilized probes, but might also promote non-specific interactions. As a result, macromolecules that are not complementary to the immobilized sequence, such as ribosomal RNA or residual genomic DNA, may adsorb to the bead surface. Such non-specific binding can interfere with downstream applications by reducing target specificity and introducing background signals.

To investigate a potential influence of the sample matrix on the viability and efficiency of the sample preparation, especially on the hybridization process, we spiked an RNA extract with a complementary oligonucleotide sequence and provided this mixture for hybridization. The investigations demonstrate that the presence of a complex real-sample matrix does not compromise the performance of the process. The comparison with a matrix-free control revealed no discernible impact on the overall outcome. The consistency of the results obtained under both conditions indicates that the process is robust against matrix-associated effects. Detailed information is shown in the supporting information, Figure S3.

## Conclusion

The presented method for sequence-specific extraction and analysis of nucleic acids using mass spectrometry holds significant promise for advancing molecular diagnostics. The successful demonstration of hybridization and digestion on magnetic beads, coupled with MALDI-TOF MS analysis, presents a robust approach for detecting specific nucleic acid sequences with high specificity and accuracy.

A key advantage of MALDI-TOF MS is its speed and minimal sample preparation requirements, especially when compared to traditional methods such as PCR. It makes the technique highly efficient and suitable for rapid diagnostics, reducing the time and resources needed for nucleic acid analysis.

Future research should focus on refining the digestion process to achieve blunt ends consistently, enhancing the method’s precision. Additionally, exploring the application of this technique to a broader range of nucleic acid targets and mutations will further validate its versatility and robustness.

In addition to the sensitivity of hybridization to mutations in the middle of the strand, the ability to distinguish between mutated and complementary strands based on mass highlights the method’s potential for precise genetic analysis. This could be valuable, particularly in clinical diagnostics, where accurate detection of genetic mutations is crucial.

Overall, this method represents a significant step forward in nucleic acid diagnostics, offering a combination of selectivity and mass accuracy, leading to more reliable and efficient detection of specific sequences in various applications.

## Supplementary Information

Below is the link to the electronic supplementary material.

Supplementary Material 1 (DOCX 286 KB)

## Data Availability

All relevant data are presented in the paper; raw data will be made available upon request.
