# Supplementary material for: Sequence-specific detection and analysis of nucleic acids via hybridization on magnetic beads and MALDI-TOF MS readout
Source: Anal Bioanal Chem. 2025 Nov 13;418(16):5143–50. doi: 10.1007/s00216-025-06193-4 (PMC13424410; doi:10.1007/s00216-025-06193-4)
Supplement: Supplementary file 1 — Supplementary Material 1 (DOCX 286 KB) [file 216_2025_6193_MOESM1_ESM.pdf]

Supporting Information

Sequence-Specific Detection and Analysis of Nucleic Acids via Hybridization on Magnetic Beads and MALDI-TOF MS Readout

Susanne Dietrich[1,2], Jessica Beyerl[2,3], Susanna Oswald[2], Anna-Cathrine Neumann-Cip[3,4], Andreas Wieser[2,3,4], Christoph Haisch[1,2]*

1: Chair of Analytical Chemistry, TUM School of Natural Sciences, Technical University of Munich, Munich, Germany

2: Fraunhofer Institute for Translational Medicine and Pharmacology ITMP, Immunology, Infection and Pandemic Research, Munich, Germany

3: Institute of Infectious Diseases and Tropical Medicine, LMU University Hospital, LMU Munich, Germany

4: German Center for Infection Research (DZIF), Partner Site Munich, Munich, Germany

***Corresponding author:**
Christoph Haisch
Lichtenbergstraße 4
85748 Garching
Germany
Haisch@tum.de
+49 89 289-154 504

1 SI Fluorescence Spectra to Confirm the Hybridization Process

The hybridization process was also evaluated by fluorescence microscopy despite MALDI-TOF MS readout. For that, the DNA fragments, which differ in up to four sequences and are available for hybridization, were labeled with the fluorescent dye Fluorescein-5,6-isothiocyanate (FITC). If hybridization occurs, fluorescence can be detected; otherwise, the sequence with the FITC is washed away, and no fluorescence can be detected. The results of the fluorescence microscope (see **Fig*ure S1***) confirm the results of the MALDI-TOF MS measurements. The sum of the 10 % most intense pixels for the image with zero mutations is 1,819,610 a.u. For two mutations in the sequence, this sum corresponds to 520,689 a.u., which corresponds to 28.6 % of the sum of the 10 % most intense pixels for the image without mutations. This sum is even lower for the fluorescence microscopic image of the sequence with 4 mutations and corresponds to 317,656 a.u.

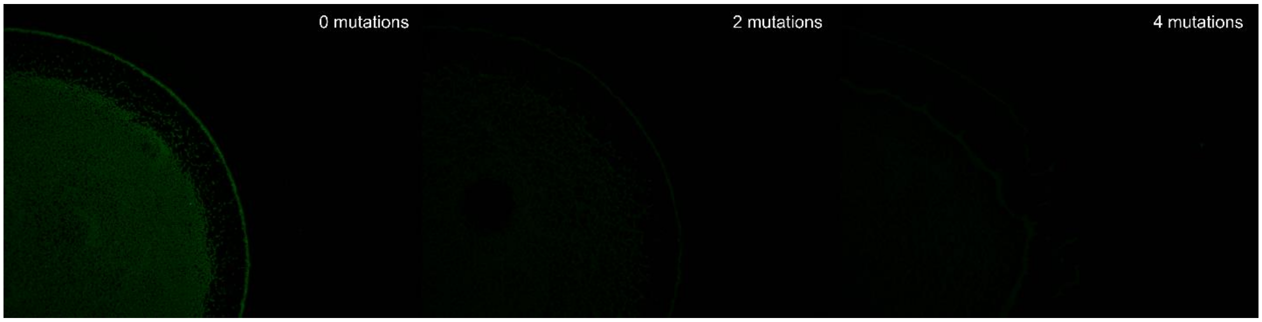

Figure S1 Fluorescence microscopic images with a GFP filter, 10× magnification, and 1/30 s exposure time of three different spots. The DNA fragments for hybridization differ in up to four nucleotides in the middle of their sequence. Left: Zero mutations in the sequence, middle: two mutations in the middle of the sequence, and right: four mutations in the middle of the sequence.

2 SI Comparison of the Digestion Products of two Sequences with Varying GC Content

To evaluate the influence of the GC content on the digestion process, two strands were investigated with varying GC content at the end of the double-stranded (ds) region.

The following sequences were used for this experiment:

- angel AT-rich: Biotin – 5' – CGC AGT AGT CTT TTT ATA TA – 3'
- cDNA AT-rich: 3' – GCG TCA TCA GAA AAA TAT ATC ACT TCT AGC TG – 5'
- angel GC-rich: Biotin – 5' – CGC AGT AGT CTT TTT GCG CG – 3'
- cDNA GC-rich: 3' – GCG TCA TCA GAA AAA CGC GCC ACT TCT AGC TG – 5'

The two strands differ in five nucleobases at the end of the ds region (black). Both strands were digested for 30 min with MB. As shown in *Figure S2*,the AT-rich strand shows more digestion products in the ds region than the GC-rich strand. Furthermore, the highest intensity in the spectrum of the GC-rich oligonucleotide is at the end of the ds region, at the beginning of the single-stranded (ss) extension (grey), where digestion should stop. In contrast, the intensities of the digestion products of the AT-rich strand are similar in height, also within the ds region.

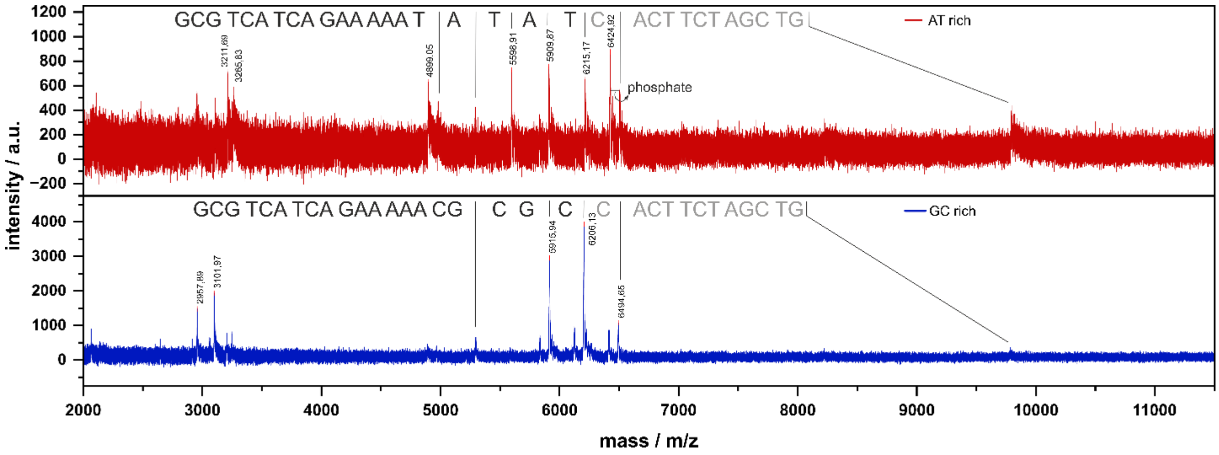

Figure S2 Comparison of two mass spectra of oligonucleotides after digestion, which are rich in AT (top) and GC (bottom), respectively. The sequence for the double-stranded region is written in black, and the sequence of the ss extension is in grey. The samples were digested for 30 min with MB.

3 SI Spiked RNA Extract with a Complementary Oligonucleotide Sequence

The matrix of a real sample can always lead to complications. In this study, we wanted to make sure that the matrix of the RNA extract has no influence on the hybridization process. We used *E. Coli* Top10 (K12), which were mixed from a stock culture with LB medium. The mixture was incubated overnight at 400 rpm at 37 °C. The RNA of *E. Coli* was extracted with the Monarch Total RNA Miniprep Kit from New England Biolabs, which purifies total RNA of all sizes. The RNA concentration, measured with a NanoDrop (NanoDrop™ 2000/2000c Spectrophotometer from Thermo Fisher Scientific), was 669 ± 43 ng/µL. For this experiment, we used the same oligonucleotides as for the digestion studies. In one aliquot, only the complementary oligonucleotide was added to the magnetic beads with immobilized sequence. To the other aliquot, the RNA extract spiked with the same amount of complementary oligonucleotide was added. As the spectra in *Figure S3* show, there is no difference if only the complementary sequence is available for hybridization or if the sequence is added in a real sample matrix (the two upper spectra). Also, the subsequent digestion process shows similar results (the two bottom spectra).

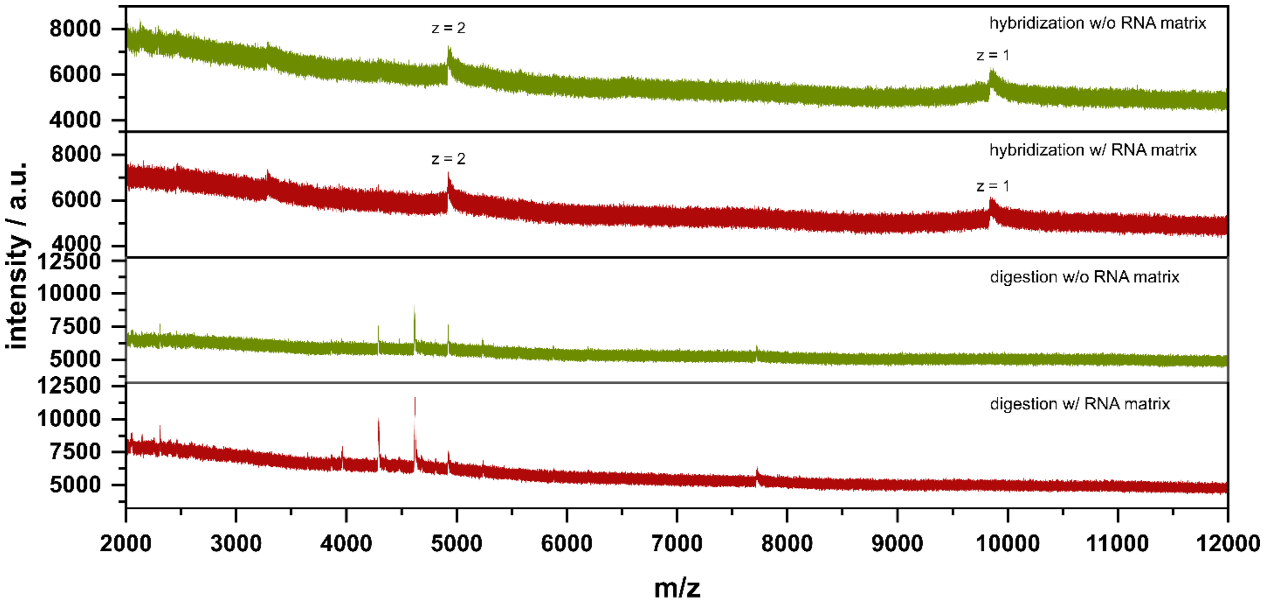

Figure S3 Mass spectrum of oligonucleotides after hybridization without RNA matrix (first) and with RNA matrix (second), and after the digestion process with S1 for 10 min and MB for 20 min without RNA matrix during the hybridization process (third) and with RNA matrix during hybridization (fourth).
